# S14G‐humanin attenuates renal ischaemia–reperfusion injury, potentially through activation of STAT3 and ERK1/2 signalling pathways in rats

**DOI:** 10.1113/EP094006

**Published:** 2026-08-31

**Authors:** Leyla Abueid, Ahmet Faruk Torun, Ezgi Golal, Nuray Acar, Filiz Basrali

**Affiliations:** ^1^ Department of Physiology, Faculty of Medicine Akdeniz University Antalya Turkey; ^2^ Department of Histology and Embryology, Faculty of Medicine Akdeniz University Antalya Turkey

**Keywords:** humanin, mitochondrial dysfunction, oxidative stress, renal ischaemia reperfusion

## Abstract

Renal ischaemia–reperfusion (I/R) injury leads to acute tubular necrosis and renal failure, triggering pathological mechanisms including inflammation, reactive oxygen species generation, apoptosis and mitochondrial dysfunction. The mitochondrial peptide humanin (HN), known to possess anti‐apoptotic and anti‐inflammatory properties, has been shown to counteract oxidative stress and restore mitochondrial function. This study aimed to investigate the effects of S14G‐humanin (humanin G, HNG) on renal I/R injury. Sprague–Dawley male rats were divided into four groups (*n* = 48): (1) Sham, (2) I/R, (3) HNG‐Sham, and (4) HNG‐I/R. In I/R groups, renal artery ligation was performed for 45 min followed by 24‐h reperfusion. HNG (2 mg/kg, i.v.) was administered 10 min before reperfusion. Urine was collected during reperfusion, and the experiment was terminated by collecting blood and tissue samples. Blood urea nitrogen and serum creatinine levels were elevated in the I/R group and were not affected by HNG treatment. Glutathione level and superoxide dismutase activity, which were diminished in the I/R group, were significantly restored following HNG administration. Myeloperoxidase activity and malondialdehyde levels were significantly decreased in HNG‐I/R group compared to the I/R group. ATP levels and mitochondrial complex I activity were significantly increased in the HNG‐I/R group compared to I/R. The percentage of apoptotic cells, markedly increased in I/R, was significantly reduced in HNG‐I/R. Signal transducer and activator of transcription 3 (STAT3) and extracellular signal‐regulated kinases 1 and 2 (ERK1/2) phosphorylation also increased in HNG‐I/R rats compared to I/R animals. HNG mitigates renal I/R injury by attenuating oxidative stress, inflammation and apoptosis while enhancing antioxidant capacity and mitochondrial function, through STAT3 and/or ERK1/2 activation.

## Abstract (highlights)

- **What is the central question of this study?**
  The mitochondria‐derived peptide humanin is recognized for its ability to enhance mitochondrial function and its anti‐apoptotic properties: what is its potential therapeutic role in acute kidney injury (a condition where mitochondrial dysfunction and apoptosis are central to the pathogenesis)?
- **What is the main finding and its importance?**
  Humanin protects against renal ischaemia–reperfusion‐induced acute kidney injury by improving mitochondrial function, enhancing antioxidant defences and reducing apoptosis. These effects may involve STAT3 and ERK1/2 activation and/or direct mitochondrial interactions. However, despite these improvements, humanin G did not improve renal function or histopathological injury, indicating cellular effects were insufficient for measurable functional or structural protection experimentally.

## 1 INTRODUCTION

Renal ischaemia–reperfusion (I/R) injury, which may occur in conditions such as haemorrhage, shock, sepsis or transplantation, can lead to acute kidney failure, which is associated with high morbidity and mortality rates (Kuwabara et al., 2022; Requião‐Moura et al., 2015). I/R injury is a process that begins during the ischaemic period and continues to increase during reperfusion (Eltzschig & Eckle, 2011). Renal ischaemia damages tubular endothelial and epithelial cells, and, if severe, can result in apoptosis and necrosis (Basile et al., 2008; Wilhelm et al., 2001). During reperfusion, oxygen is resupplied, but this also causes increased formation of reactive oxygen species (ROS) (Malek & Nematbakhsh, 2015). Increased ROS production exacerbates tissue damage through leukocyte activation and migration, peroxidation of membrane lipids and oxidative damage to intracellular structures, thereby worsening the degree of apoptosis and necrosis (Li et al., 2022).

Mitochondria play a crucial role in ATP synthesis, energy metabolism, regulation of apoptosis and Ca^2+^ homeostasis (Bhargava & Schnellmann, 2017). Mitochondrial dysfunction observed in renal I/R injury is characterized by mitochondrial swelling, expansion of cristae and intermembrane spaces, and a reduction in mitochondrial membrane potential (Su et al., 2023). Damaged mitochondria during I/R inhibit the activity of the electron transport chain (ETC) enzymes, including complex I, complex II and ATP synthase, resulting in significant electron leakage, hence the increased ROS production (Stanley, 2004). It is well known that the balance between the oxidant and antioxidant systems is maintained under physiological conditions, and this balance is disrupted in I/R injury due to both increased ROS production and a weakened antioxidant system (Johnson & Weinberg, 1993). Various antioxidant agents, such as catalase (CAT), superoxide dismutase (SOD) and glutathione (GSH), have been shown to mitigate ROS‐mediated injury during renal I/R. Evidence from experimental kidney I/R models indicates that oxidative stress‐related tissue damage is strongly linked to diminished GSH levels (Basile et al., 2012; Singh et al., 1993).

Humanin (HN) is a mitochondria‐derived peptide identified first in amyloid‐beta plaques of Alzheimer's patients (Hashimoto et al., 2001; Tajima et al., 2002). It is known to improve mitochondrial functions, enhance the antioxidant system and have an anti‐apoptotic effect (Guo et al., 2003; Sreekumar et al., 2016). It has been shown that HN exhibits protective and therapeutic effects against I/R injury in cerebral, myocardial and intestinal tissues (Abozaid et al., 2023; Kumfu et al., 2016; Zhao et al., 2012). Additionally, it has been demonstrated that HN exerts its cytoprotective and anti‐apoptotic effects under cellular stress conditions in various cell types (Gao et al., 2017; Hazafa et al., 2021). Although the intracellular signalling mechanisms underlying the effects of HN have not yet been fully elucidated, current evidence highlights the signal transducer and activator of transcription 3 (STAT3) and extracellular signal‐regulated kinase 1 and 2 (ERK1/2) pathways as key mediators of its cytoprotective and anti‐apoptotic effects (Gao et al., 2017; Kim et al., 2016). Various preconditioning and postconditioning interventions that attenuate I/R injury have been shown to activate both STAT3 and ERK1/2 signalling pathways. During reperfusion, levels of proinflammatory cytokines such as interleukin (IL)‐6 and tumour necrosis factor (TNF)‐α rise rapidly, triggering downstream signalling cascades involving pathways such as Janus kinase (JAK)/STAT3 and ERK1/2 (Yang et al., 2023). ERK1/2, a member of the mitogen‐activated protein kinase (MAPK) pathway, is involved in many cellular processes including cell proliferation, survival, differentiation and apoptosis, and can be activated by ROS and pro‐inflammatory mediators, especially during reperfusion (Yu et al., 2023). Although overstimulation of ERK1/2 signalling can contribute to inflammation, a certain level of ERK1/2 activation is crucial for the induction of cell survival, proliferation and repair signalling during I/R injury, as it is known to be involved in reperfusion injury salvage kinase (RISK) signalling (Yang et al., 2023; Yu et al., 2023). On the other hand, STAT3 activation in intrinsic protective pathways (such as the SAFE pathway) has been associated with improved mitochondrial function and reduced infarct size in cardiac I/R injury models (Heusch et al., 2011). This coordinated activation appears to represent an adaptive compensatory response that limits apoptosis, preserves mitochondrial function and restrains excessive inflammatory signalling (Yang et al., 2023).

A potent humanin analogue, S14G‐humanin (humanin G, HNG), is generated by replacement of Ser14 with glycine, and this substitution potently increases humanin activity (Hashimoto et al., 2001). The present study used HNG to evaluate its potential protective or therapeutic effects against renal I/R injury *in vivo* and to identify the potential mechanism/s underlying this protection. Although there are few studies examining the effects of HNG in experimental chronic renal failure models, its acute effects on renal I/R injury, which is accepted as an experimental model of acute kidney injury (AKI) has not yet been investigated.

In the present study, we aimed to investigate the effects of acute HNG administration and the underlying mechanisms in a rat model of AKI induced by renal I/R.

## 2 METHODS

### 2.1 Ethical approval

All experimental procedures involving rats were conducted in accordance with the policies and regulations of *Experimental Physiology* regarding animal experimentation and complied with the guidelines established by the Animal Care and Usage Committee of Akdeniz University. The experimental protocol was approved by the Animal Care and Usage Committee of Akdeniz University (Approval No. 2020.08.004‐2024.04.007).

### 2.2 Animals and chemicals

Forty‐eight male Sprague–Dawley rats aged 8–10 weeks, weighing 200–250 g were used in this study. Rats were obtained from the Akdeniz University Experimental Animals Application and Research Center (Antalya, Türkiye). Animals were housed under standard laboratory conditions with free access to standard chow and water throughout the study. They were maintained under a 12‐h light–dark cycle in a controlled environment with a temperature of 22 ± 2°C and relative humidity ranging from 60% to 70%. During the experimental period, four animals were lost during the perioperative period, including two animals from the ischaemia–reperfusion (I/R) groups due to surgical complications and two animals from the sham groups. Consequently, the number of animals available for subsequent analyses varied among the experimental groups. In addition, because multiple biochemical, histological and molecular analyses were performed using tissues obtained from the same animals, the available kidney tissues were allocated among the different experimental procedures according to tissue availability. The number of samples included in certain assays was also limited by assay kit availability. Therefore, the sample size (*n*) differs between analyses and is indicated in the corresponding tables and figure legends. In all analyses, *n* represents the number of individual animals analysed for the respective endpoint.

HNG (S14G‐humanin; amino acid sequence: Met‐Ala‐Pro‐Arg‐Gly‐Phe‐Ser‐Cys‐Leu‐Leu‐Leu‐Leu‐Thr‐Gly‐Glu‐Ile‐Asp‐Leu‐Pro‐Val‐Lys‐Arg‐Ala) was purchased from GenScript Biotech Corporation (Piscataway, NJ, USA) and used as a HN analogue.

### 2.3 Induction of renal I/R injury

Rats were randomly divided into four groups: Sham (*n* = 12), ischemia–reperfusion (I/R; *n* = 12), humanin G‐sham (HNG‐Sham; *n* = 12) and HNG‐I/R (*n* = 12). The Sham groups did not undergo I/R procedures, whereas the IR groups were subjected to 45 min of bilateral renal ischaemia followed by a 24‐h reperfusion period.

All rats were anesthetized with intraperitoneal (i.p.) ketamine/xylazine (90/10 mg/kg). The tail vein of each rat was cannulated with a 24‐G cannula for injections. In the sham groups, a midline abdominal incision was made to expose the renal artery and vein without applying the I/R protocol (Wilhelm et al., 2001).

In the I/R groups, bilateral renal ischaemia was induced by clamping both renal pedicles with atraumatic vascular clamps for 45 min, followed by reperfusion. In the HNG‐Sham and HNG‐I/R groups, HNG was administered intravenously via the tail vein at a dose of 2 mg/kg, 10 min before the reperfusion period. Upon completion of the ischaemic period, the clamps were removed and the incision was ligated. The incision site was disinfected with povidone‐iodine after the operation. During the 24‐h reperfusion period, the animals were housed individually in metabolic cages with free access to food and water while their urine samples were collected (Wilhelm et al., 2001). At the end of reperfusion, animals were euthanized by exsanguination under deep anaesthesia while blood samples and kidney tissues were collected for further analysis in accordance with institutional guidelines.

### 2.4 Assessment of renal function

Blood samples (0.5 mL) were collected and centrifuged to obtain supernatants. Serum creatinine (sCr) and blood urea nitrogen (BUN) levels were assessed using an autoanalyser (Cobas c502; Roche Diagnostics, Rotkreuz, Switzerland) at the Central Laboratory of Akdeniz University Hospital. Creatinine clearance (ClCr) as an indicator of glomerular filtration rate (GFR) was calculated using the following formula (Tobias et al., 1962):

$$\begin{array}{ccc} \mathrm{ClCr}\;\left(\mathrm{mL}/\min\right) & = & \;\mathrm{Urine}\;\mathrm{creatine}\;\left(\mathrm{mg}/\mathrm{dL}\right)\;\times \;\mathrm{Urine}\;\mathrm{flow}\;\left(\mathrm{mL}/\min\right)/\\ & & \mathrm{Serum}\;\mathrm{creatine}\;\left(\mathrm{mg}/\mathrm{dL}\right) \end{array}$$

### 2.5 Histopathological assessment

The formalin‐fixed and paraffin‐embedded kidney was cut into 5 µm sections and stained with haematoxylin and eosin (H&E) for histological assessment. Histological injury scores were determined by assessing tubular necrosis, loss of the brush border, tubular cast formation and tubular dilation as described previously (Tighe, 1977). The histological damage scores were counted in a blind study by two observers.

### 2.6 Apoptosis assessment

Apoptotic cells in rat kidney tissue were evaluated using a terminal deoxynucleotidyl transferase deoxyuridine triphosphate (dUTP) nick end labelling (TUNEL) assay kit (Abcam, Cambridge, UK; cat. no. ab206386) in accordance with the manufacturer's instructions. TUNEL‐stained sections were evaluated using a light microscope (Zeiss Microscopy, Oberkochen, Germany) with a special ocular scale. Five randomly selected slides, each with four different fields at ×10 magnification, were evaluated for the analysis of TUNEL. Positively stained cell numbers in the fields were counted in a blind study by two observers. Mean positive cell numbers were calculated and then were rated to 100. Positively stained apoptotic cell numbers (apoptotic index) in the tissue were indicated as a percentage.

### 2.7 Western blot analysis

For western blot analysis, kidney tissues obtained from four animals per group were used to prepare a total of six homogenates. The protein concentration of each sample was determined using a bicinchoninic acid (BCA) assay kit. Equal amounts of protein (20 µg/mL) were loaded into each well and separated by 10% SDS‐PAGE at 100 V and 30 mA for 2 h. The separated proteins were then transferred to a polyvinylidene fluoride membrane using a transfer system (Trans‐Blot Turbo, Bio‐Rad Laboratories, Hercules, CA, USA) at 25 V and 1.3 A for 10 min.

The membranes were incubated overnight with primary antibodies for p‐STAT3 (cat. no. 9145T, Cell Signaling Technology, Danvers, MA, USA; RRID:AB_2491009) at 1:1000 dilution, p‐ERK1/2 (cat. no. 4370T, Cell Signaling, RRID:AB_2315112) at 1:1000 dilution, and β‐actin. A Goat anti‐rabbit IgG conjugated secondary antibody was used in accordance with the primary antibodies. The membranes were incubated with a chemiluminescent substrate for 5 min, and the bands were visualized using an Azure C280 imaging system (Azure Biosystems, Dublin, CA, USA). Proteins on the p‐STAT3 and p‐ERK1/2 membranes were then incubated overnight with the β‐actin antibody (cat. no. 4970, Cell Signaling, RRID:AB_2223172) at a dilution of 1:5000, served as an internal control. The obtained results were analysed using ImageJ software (v. 1.36b; National Institutes of Health, Bethesda, MD, USA, RRID:SCR_003070) and protein expression was quantified by normalizing the target protein to the reference protein.

### 2.8 Assessments of mitochondrial functions

#### 2.8.1 Intracellular ATP levels

ATP levels in tissue homogenates were quantified using an ATP Assay Kit (Elabscience, Wuhan, China, cat. no. E‐BC‐K157‐S) following the manufacturer's instructions and expressed as ATP content per gram of tissue.

#### 2.8.2 Complex I activities

Complex I activity in kidney tissue homogenates was determined using a Complex I Activity Assay Kit (Abcam, cat. no. ab109721), following the manufacturer's protocol. Results were expressed as enzyme activity per milligram of tissue.

### 2.9 Assessments of oxidative stress

Malondialdehyde (MDA) levels, which are indicators of lipid peroxidation, were determined in kidney tissue homogenate by the reaction of MDA with thiobarbituric acid (TBA) (Sigma‐Aldrich, St Louis, MO, USA, cat. no. T5500) and detected using spectrophotometric methods as described previously (Buege & Aust, 1978). MDA levels were expressed as nmol MDA/g tissue.

### 2.10 Assessments of antioxidant status

GSH levels, SOD and CAT activity were examined as antioxidant parameters in kidney tissue homogenates.

The level of endogenous antioxidant GSH in tissue was measured by modifying the Ellman method, which detects sulfhydryl groups (Aykaç et al., 1985; Ellman, 1959). Absorbances were measured on a Shimadzu UV‐1601 spectrophotometer (Shimadzu Corporation, Kyoto, Japan) (at 412 nm), and the calculated GSH level was expressed as µmol/g tissue.

SOD activity was determined using a previously described method (Masayasu & Hiroshi, 1979). The superoxide radical generated by riboflavin under fluorescent light (20 W) is converted to hydrogen peroxide (H_2_O_2_) by SOD and subsequently H_2_O_2_ is detected using *o*‐dianisidine hydrochloride. SOD activity was calculated from the difference between reaction absorbance and basal absorbance at 460 nm in kidney tissue and was expressed as U/g tissue (Mylroie et al., 1986).

CAT activity in kidney tissue homogenates was determined using spectrophotometric methods as previously described by Aebi (1984). Absorbance was measured at 240 nm using a spectrophotometer (Shimadzu UV‐1601). CAT activity was calculated based on an extinction coefficient of 0.004 mM^−^^1^ mm^−^^1^ and expressed as U/g tissue.

### 2.11 Assessments of inflammation

Kidney tissue myeloperoxidase (MPO) activity was evaluated as an indicator of inflammation. MPO activity was determined through the reaction of H_2_O_2_ oxidation with *o*‐dianisidine HCl (Bradley et al., 1982). Absorbance changes due to the reaction were measured at 460 nm using a spectrophotometer (Shimadzu UV‐1601). MPO activity was expressed as U/g tissue.

### 2.12 Statistical analysis

Statistical analyses were performed using GraphPad Prism version 10.6.1 (GraphPad Software Inc., Boston, MA, USA, RRID:SCR_002798). Data are presented as means ± standard deviation (SD). Data normality was assessed using the Shapiro–Wilk test. Normally distributed variables were analysed using one‐way analysis of variance (ANOVA) followed by Tukey's multiple comparisons test. Histopathological scores were analysed using the Kruskal–Wallis test followed by Dunn's multiple comparisons test. *P* < 0.05 was considered statistically significant.

## 3 RESULTS

### 3.1 HNG fails to restore renal function following renal I/R injury

As shown in Table 1, rats in the I/R groups displayed significant deterioration of renal function 24 h after reperfusion, as reflected by increased levels of sCr and BUN with decreased creatine clearance (ClCr, an indicator of GFR) compared to those in sham groups. Furthermore, sCr, BUN and ClCr levels in the HNG‐I/R group did not differ significantly from those in the I/R group (the *P*‐values were 0.884, 0.993 and 0.763, respectively), indicating that HNG treatment did not improve renal functional parameters under the conditions of this study.

**TABLE 1:** Effects of HNG treatment on renal functions.

	Sham	I/R	HNG‐Sham	HNG‐I/R
**sCr (mg/dL)**	0.340 ± 0.08	1.422 ± 1.01***	0.357 ± 0.11	1.227 ± 0.68**
**BUN (mg/dL)**	18.83 ± 1.7	70.20 ± 33.6^***^	19.00 ± 2.7	73.11 ± 37.6^***^
**ClCr (mL/min)**	1.065 ± 0.3	0,35 ± 0.27^***^	0.93 ± 0.33	0.506 ± 0.50

Note: Data are presented as means ± SD (n = 10). Comparisons were performed between each ischaemia–reperfusion group and its corresponding sham group (I/R vs. Sham; HNG‐I/R vs. HNG‐Sham). Serum creatinine (sCr) was significantly higher in the I/R group than in the Sham group (P < 0.001) and in the HNG‐I/R group than in the HNG‐Sham group (P = 0.00995). Blood urea nitrogen (BUN) was significantly higher in the I/R group than in the Sham group (P < 0.001) and in the HNG‐I/R group than in the HNG‐Sham group (P < 0.001). Creatinine clearance (ClCr) was significantly lower in the I/R group than in the Sham group (P < 0.001), whereas the difference between the HNG‐I/R and HNG‐Sham groups was P = 0.053. Comparisons between the HNG‐I/R and I/R groups yielded P‐values of 0.884 for sCr, 0.993 for BUN and 0.763 for ClCr. **P = 0.009, ***P < 0.001.

Abbreviations: BUN, blood urea nitrogen; ClCr, creatinine clearance; HNG, humanin G; I/R, ischaemia–reperfusion; sCr, serum creatinine.

### 3.2 HNG treatment attenuates apoptosis without ameliorating histopathological damage in renal I/R injury

Renal tissues were examined after H&E staining (Figure 1a). Structural changes in the tubules, such as tubular necrosis, atrophy, dilation in the tubules and Bowman's capsule, loss of the brush border and cast formation, were observed and histopathologically scored (Figure 1b). The histopathological damage score in the I/R group was significantly higher compared to the Sham group (*P *< 0.001). The histopathological damage score in the HNG‐I/R group was also significantly higher compared to the HNG‐Sham group (*P = *0.00691). The damage observed in the HNG‐I/R group was not significantly different from that in the I/R group.

**FIGURE 1:**
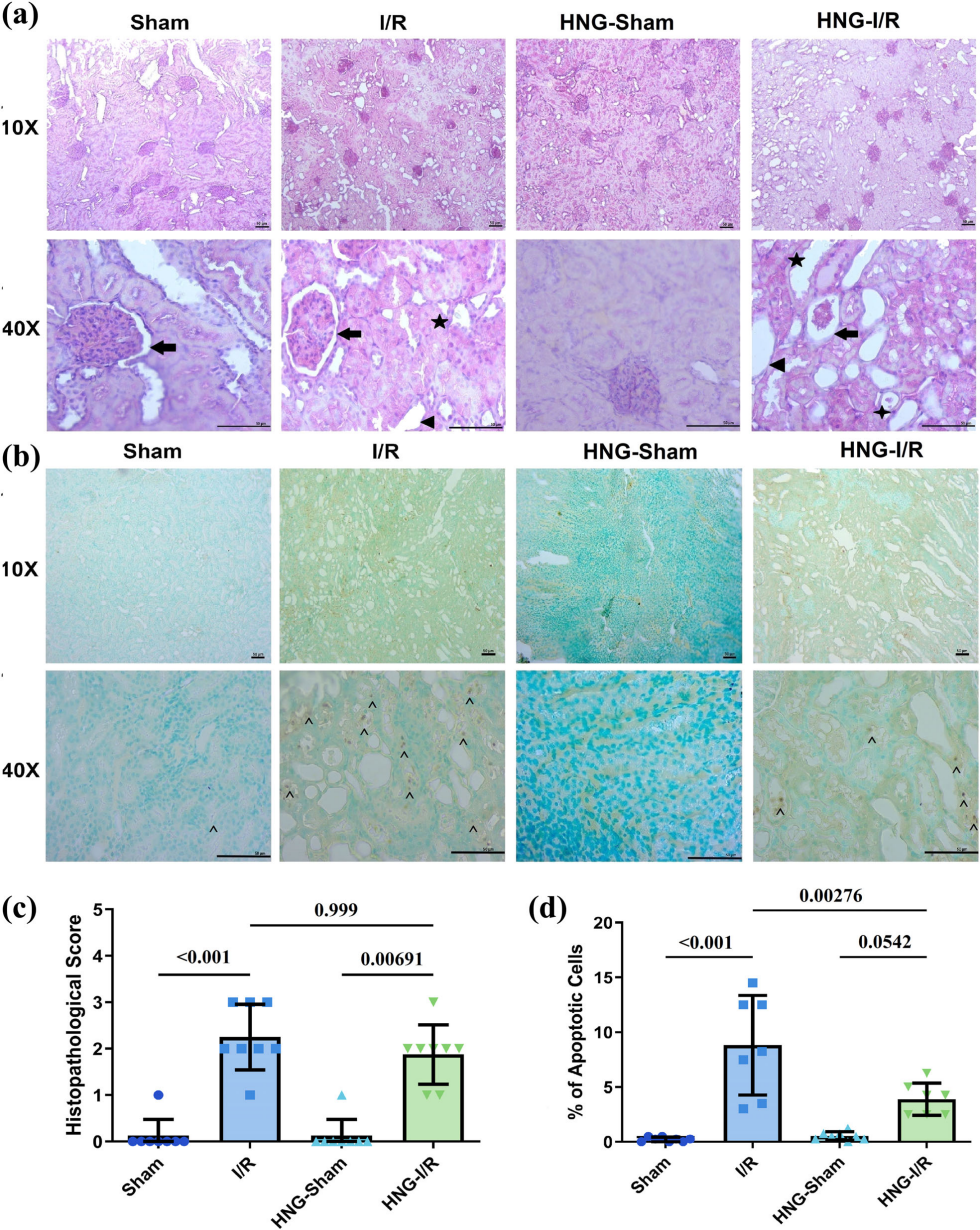
Histopathological damage and apoptosis in renal tissue after I/R injury and HNG treatment. (a) Representative H&E‐stained kidney sections showing tubular necrosis (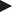), tubular dilation (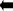), cast formation (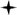) and loss of brush border (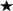) in experimental groups. (b) Representative TUNEL‐stained kidney sections showing apoptotic cells (brown‐stained nuclei). (c) Semi‐quantitative histopathological injury scores based on tubular necrosis, tubular dilation, cast formation and brush border loss (*n* = 8). Sham vs. I/R, *P *< 0.001; HNG‐Sham vs. HNG‐I/R, *P* = 0.00691; I/R vs. HNG‐I/R, *P* = 0.999. (d) Quantification of apoptotic index (% TUNEL‐positive cells) (*n* = 7). Sham vs. I/R, *P *< 0.001; I/R vs. HNG‐I/R, *P* = 0.00276; HNG‐Sham vs. HNG‐I/R, *P* = 0.0542. Data are presented as means ± SD. Exact *P* values for *post hoc* comparisons are shown above the bars. Scale bars: 50 µm. HNG, humanin G; I/R, ischaemia–reperfusion; TUNEL, terminal deoxynucleotidyl transferase dUTP nick end labelling.

Apoptotic cells in renal tissues were detected immunohistochemically using the TUNEL kit (Figure 1b). Apoptotic cells were counted, and the percentage of apoptotic cells was calculated (Figure 1d). The percentage of apoptotic cells in the I/R group was significantly higher compared to the Sham group (*P* < 0.001). The apoptotic cell density in the HNG‐I/R group was significantly lower compared to the I/R group (*P =* 0.00276). No significant difference was observed between the HNG‐Sham and HNG‐I/R groups (*P* = 0.0542).

### 3.3 HNG improves mitochondrial function disrupted by renal I/R injury

To evaluate mitochondrial functions, intracellular ATP levels in renal tissue homogenates were measured (Figure 2a). The ATP levels in the I/R group were found to be significantly lower compared to the Sham group (*P* < 0.001). Tissue ATP levels measured in the HNG‐I/R group were significantly elevated compared to the I/R group (*P = *0.0414). No significant difference was observed between the HNG‐Sham and HNG‐I/R groups (*P* = 0.550).

**FIGURE 2:**
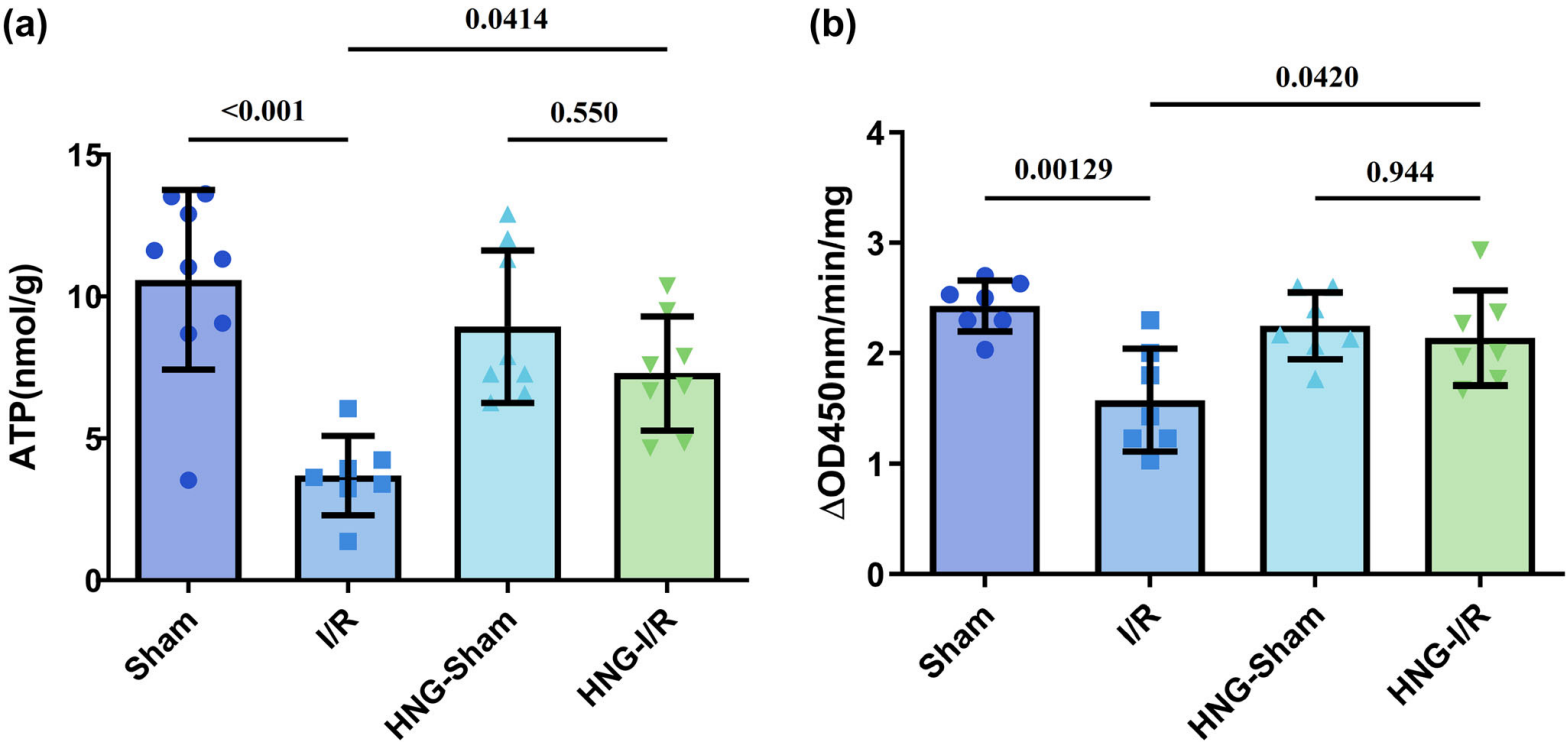
Effects of HNG on mitochondrial function in renal tissue following I/R injury. (a) Intracellular ATP levels in renal tissue homogenates (*n* = 8). Sham vs. I/R, *P* < 0.001; I/R vs. HNG‐I/R, *P* = 0.0414; HNG‐Sham vs. HNG‐I/R, *P* = 0.550. (b) Mitochondrial complex I activity in renal tissue homogenates (*n* = 7). Sham vs. I/R, *P* = 0.00129; I/R vs HNG‐I/R, *P* = 0.0420; HNG‐Sham vs HNG‐I/R, *P* = 0.944. Data are presented as means ± SD. Exact *P*‐values for *post hoc* comparisons are shown above the bars. HNG, humanin G; I/R, ischaemia–reperfusion.

As an indicator of mitochondrial function, complex I activity levels were measured in renal tissue homogenates (Figure 2b). The complex I activity in the I/R group was significantly lower compared to the Sham group (*P = *0.00129). However, the complex I activity levels in the HNG‐I/R group were significantly higher compared to the I/R group (*P = *0.0420). No significant difference was observed between the HNG‐Sham and HNG‐I/R groups (*P* = 0.944).

### 3.4 HNG attenuates oxidative stress and restores antioxidant capacity following renal I/R injury

Levels of MDA, which is the product of lipid peroxidation in renal tissue homogenates, are presented in Figure 3a. MDA levels were significantly higher in the I/R group compared to the Sham group (*P* < 0.001). In the HNG‐I/R group, MDA levels were not significantly different from those in the HNG‐Sham group (*P = *0.258). However, tissue MDA levels were significantly lower in the I/R group treated with HNG compared to the untreated group (*P = *0.0150).

**FIGURE 3:**
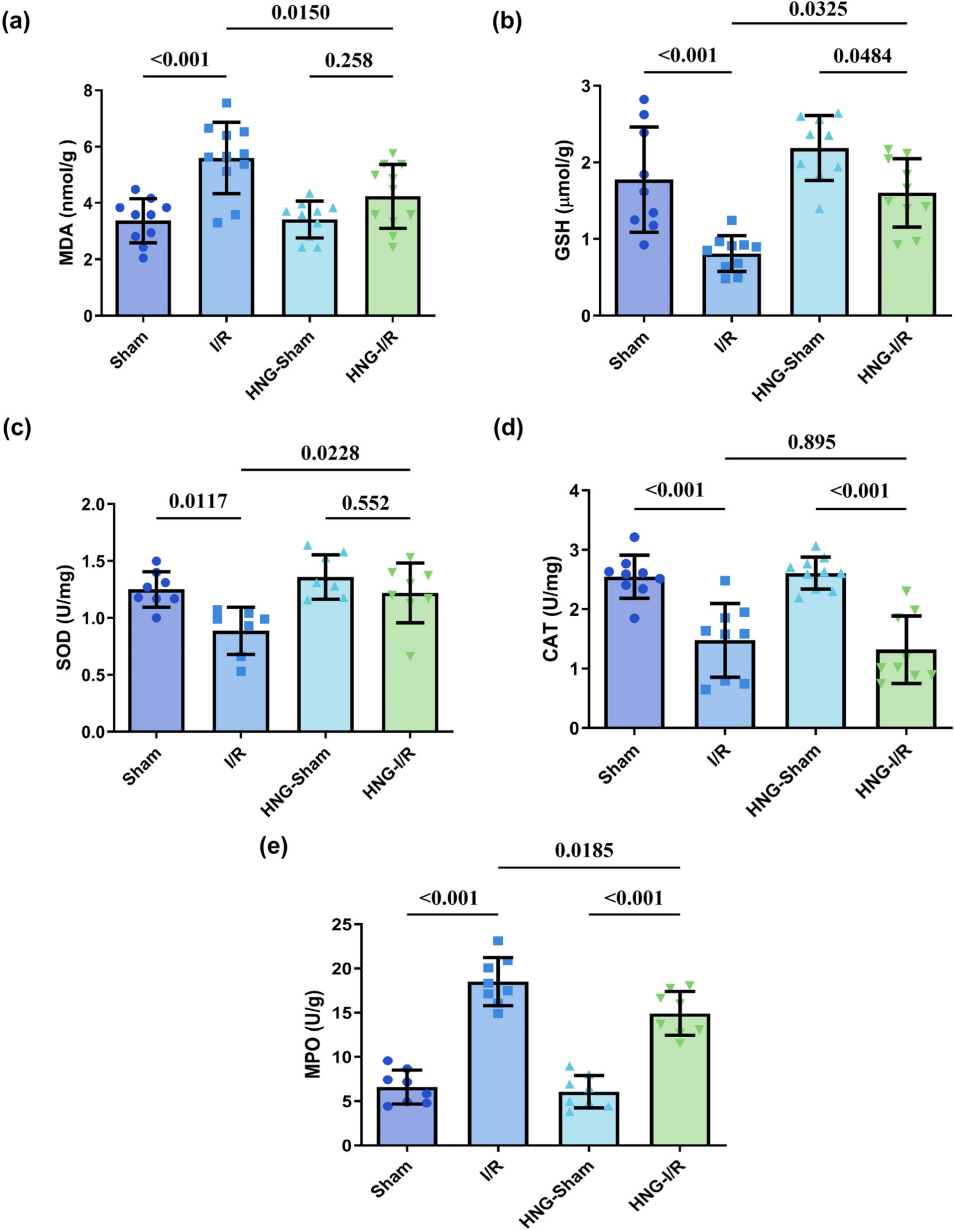
Effects of HNG on oxidative stress, antioxidant status and inflammation in renal tissue. (a) Malondialdehyde (MDA) levels (*n* = 11). Sham vs. I/R, *P *< 0.001; I/R vs. HNG‐I/R, *P *= 0.0150; HNG‐Sham vs. HNG‐I/R, *P *= 0.258). (b) Glutathione (GSH) levels (*n* = 10). Sham vs. I/R, *P *< 0.001; I/R vs. HNG‐I/R, *P *= 0.00325; HNG‐Sham vs. HNG‐I/R, *P *= 0.0484. (c) Superoxide dismutase (SOD) activity (*n* = 10). Sham vs. I/R, *P *= 0.0117; I/R vs. HNG‐I/R, *P *= 0.0228; HNG‐Sham vs. HNG‐I/R, *P *= 0.552. (d) Catalase (CAT) activity (*n* = 10). Sham vs. I/R, *P *< 0.001; HNG‐Sham vs. HNG‐I/R, *P *< 0.001; I/R vs. HNG‐I/R, *P* = 0.895. (e) Myeloperoxidase (MPO) activity (*n* = 8). Sham vs. I/R, *P *< 0.001; HNG‐Sham vs. HNG‐I/R, *P *< 0.001; I/R vs. HNG‐I/R, *P *= 0.0185. Data are presented as means ± SD. Exact *P*‐values for *post hoc* comparisons are shown above the bars. CAT, catalase; GSH, glutathione; HNG, humanin G; I/R, ischaemia–reperfusion; MDA, malondialdehyde; MPO, myeloperoxidase; SOD, superoxide dismutase.

Renal tissue GSH levels, as shown in Figure 3b, were significantly reduced in the I/R group compared to the Sham group (*P < *0.001). In the HNG‐I/R group, GSH levels did not differ significantly from those in the HNG‐Sham group (*P =* 0.484). However, renal GSH levels in the HNG‐I/R group were significantly higher than those observed in the I/R group (*P =* 0.00325).

SOD activity, the most effective endogenous antioxidant enzyme in cellular defence, was measured as an indicator of antioxidant capacity in renal tissue homogenates (Figure 3c). SOD activity in the I/R group was significantly lower compared to the Sham group (*P =* 0.0117). In the HNG‐I/R group, SOD activity was not significantly different from that in the HNG‐Sham group (*P = *0.552). However, renal SOD activity in the HNG‐I/R group treated with HNG prior to reperfusion was significantly higher compared to the I/R group (*P =* 0.0228).

CAT activity was measured as an antioxidant marker in renal tissue homogenates (Figure 3d). CAT activity in the I/R group was significantly lower compared to the Sham group (*P* < 0.001). Similarly, CAT activity in the HNG‐I/R group was significantly lower compared to the HNG‐Sham group (*P* < 0.001). No significant difference was found in CAT activity between the HNG‐I/R group and the I/R group (*P = *0.895).

### 3.5 HNG attenuates inflammation induced by renal I/R injury

MPO, as a marker of neutrophil infiltration into the damaged tissue, was assessed to evaluate the inflammatory response. It was significantly elevated in the I/R group compared to the Sham group (*P* < 0.001) (Figure 3e). Similarly, MPO activity in the HNG‐I/R group was significantly higher compared to the HNG‐Sham group (*P* < 0.001). Moreover, MPO activity in the HNG‐I/R group was significantly reduced compared to the I/R group, indicating a potential attenuation of the inflammatory response (*P =* 0.0185).

### 3.6 HNG increases STAT3 and ERK1/2 phosphorylation following renal I/R injury

Western blot analyses demonstrated significant alterations in ERK1/2 and STAT3 signalling among the experimental groups (Figure 4a,c). ERK1/2 phosphorylation was increased in the I/R group compared to the Sham group; however, this difference was not statistically significant (*P = *0.772). In contrast, phosphorylated ERK 1/2 (p‐ERK1/2) levels were significantly higher in the HNG‐I/R group compared with both the HNG‐Sham and I/R groups (*P* < 0.001).

**FIGURE 4:**
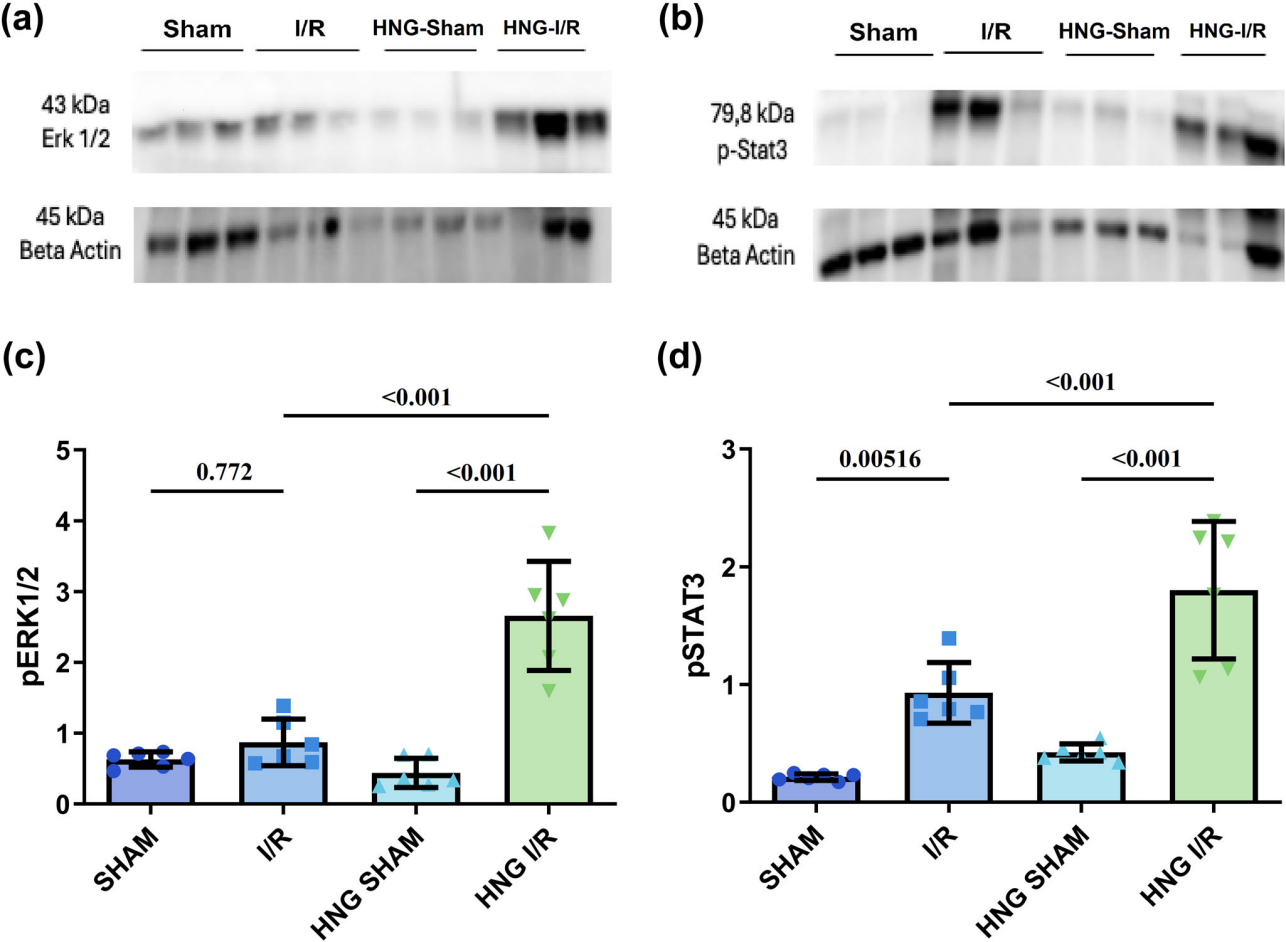
Effects of HNG Treatment on p‐ERK1/2 and p‐STAT3 phosphorylation following I/R injury. (a, b) Representative western blot bands for p‐ERK1/2, p‐STAT3 and β‐actin (45 kDa, loading control) in Sham, I/R, HNG‐Sham and HNG‐I/R groups. (c) Densitometric quantification of p‐ERK1/2 protein levels relative to β‐actin (*n* = 4 animals per group, 6 homogenates total). Sham vs. I/R, *P *= 0.772; I/R vs. HNG‐I/R, *P *< 0.001; HNG‐Sham vs. HNG‐I/R, *P *< 0.001. (d) Densitometric quantification of p‐STAT3 protein levels relative to β‐actin (*n* = 4 animals per group, 6 homogenates total). Sham vs. I/R, *P *= 0.00516; I/R vs. HNG‐I/R, *P *< 0.001; HNG‐Sham vs. HNG‐I/R, *P *< 0.001. Data are presented as means ± SD. Exact *P*‐values for *post hoc* comparisons are shown above the bars. ERK1/2, extracellular signal‐regulated kinases 1 and 2; HNG, humanin G; I/R, ischaemia–reperfusion; STAT3, signal transducer and activator of transcription 3.

Similarly, phosphorylated STAT3 (p‐STAT3) expression was significantly increased in the I/R group compared to the Sham group (*P =* 0.00516). p‐STAT3 levels in the HNG‐I/R group were markedly elevated compared with both the I/R and HNG‐Sham groups (*P* < 0.001) (Figure 4b,d). β‐Actin expression did not differ among groups, confirming equal protein loading. These results indicate that HNG treatment is associated with increased activation of ERK1/2 and STAT3 signalling pathways in renal tissue following I/R injury.

## 4 DISCUSSION

Renal I/R injury frequently arises from kidney transplantation, haemorrhage and nephrectomy and remains a significant unresolved challenge in clinical practice. It is one of the main causes of AKI and leads to disease with high mortality and morbidity (Basile et al., 2012). During I/R injury, tissue damage initiated during the ischaemic phase persists and is further exacerbated upon reperfusion (Sharfuddin & Molitoris, 2011). The main mechanism of renal I/R injury is associated with multiple mediators including oxidative stress, inflammation and mitochondrial dysfunction also with activation of adhesion molecules, ultimately resulting in renal tubular damage and apoptosis (Su et al., 2023; Thurman, 2007; Versteilen et al., 2004). At present, strategies targeting apoptosis and oxidative stress may contribute to maintaining renal function and reducing tubular injury (Havasi & Borkan, 2011).

In the present study, we aimed to investigate the effects of HNG on renal I/R injury and to elucidate the potential mechanisms involved, using HNG as an HN analogue. To the best of our knowledge, this is the first study to examine the effects of HNG administration on renal I/R injury. We found that HNG administration attenuated the pathological alteration in renal I/R injury, which was accompanied by reduced inflammation, oxidative stress and apoptosis. Additionally, HNG preserved mitochondrial function, increased complex I activity and elevated ATP levels. Together, these findings indicate that HNG exerts beneficial cellular and molecular effects following renal I/R injury in rats. Furthermore, the observed increases in STAT3 and ERK1/2 phosphorylation suggest that these signalling pathways may be associated with the cellular responses induced by HNG.

In I/R‐induced AKI, the decline in GFR is accompanied by an increase in circulating nitrogenous waste products, ultimately resulting in impaired renal function (Malek & Nematbakhsh, 2015). Although our findings confirmed the development of renal dysfunction following I/R injury, HNG treatment did not significantly improve the reduction in GFR or the elevations in sCr and BUN, the principal indicators of renal dysfunction. These findings indicate that, under the present experimental conditions, the molecular and biochemical effects of HNG were not accompanied by measurable functional recovery within 24 h of reperfusion. Whether longer reperfusion periods or alternative treatment regimens would result in improved renal function remains to be determined.

In our study, renal tissue injury was assessed through histopathological examination and apoptosis. It is well known that necrosis is the primary pathway of cell death during ischaemia in renal I/R injury (Venkatachalam et al., 2015). Although tubular necrosis scores were significantly increased in the I/R group, HNG treatment did not significantly reduce these histopathological changes. This lack of histological improvement should be considered a genuine finding of the present study. Notably, despite the absence of a reduction in tubular necrosis, HNG significantly improved mitochondrial functions, attenuated oxidative stress and inflammation and enhanced pro‐survival signalling pathways. These findings suggest that preservation of mitochondrial function and modulation of cellular stress responses alone may not be sufficient to reverse established structural renal injury within the first 24 h after severe ischaemia–reperfusion. Whether these molecular effects could translate into histological recovery after longer reperfusion periods or with alternative treatment regimens warrants further investigation.

Accumulating evidence has suggested that apoptosis is critically involved in the pathological process of renal I/R injury (Havasi & Borkan, 2011; Versteilen et al., 2004). The present study also demonstrated that I/R caused a significant increase in TUNEL‐positive tubular cells, indicating the occurrence of apoptosis. Moreover, the findings of this present study revealed that HNG treatment prior to reperfusion significantly reduced the apoptosis in renal tubular cells. Anti‐apoptotic effects of HNG have been demonstrated in numerous in vitro and in vivo studies, including I/R injury models (Abozaid et al., 2023; Guo et al., 2003). In the present study, HNG treatment was also associated with increased phosphorylation of STAT3 and ERK1/2. In light of these findings, it could be hypothesized that the anti‐apoptotic effects of HNG may be associated with STAT3 and/or ERK1/2 phosphorylation, although this requires further investigation. Further studies using specific pharmacological inhibitors or genetic approaches are required to determine the mechanistic contribution of these pathways.

Disruption of cellular energy homeostasis, particularly due to compromised mitochondrial activity, represents a central mechanism in the pathogenesis of renal I/R injury (Bhargava & Schnellmann, 2017; Su et al., 2023). It is well established that renal I/R injury induces mitochondrial dysfunction, including a reduction in ETC enzyme activity and ATP production (Ishimoto & Inagi, 2015). This phenomenon plays a crucial role in I/R induced damage in tubular epithelial cells, which are characterized by increased mitochondrial density. Previous studies have suggested that HNG increases intracellular ATP levels in various cell types including neurons, muscle cells and pancreatic β‐cells consistent with our findings (Cohen et al., 2015; Kuliawat et al., 2013; Voigt & Jelinek, 2016). The results of the present study also demonstrated that HNG treatment restored intracellular ATP levels and complex I activity, both of which were reduced by I/R injury. p‐STAT3 has been shown to enhance ATP production in hepatic I/R injury by increasing the activity of mitochondrial respiratory chain complexes I and II (Yang et al., 2023). In our study, the observed increase in ATP levels and complex I activity following HNG treatment may have been mediated by p‐STAT3‐dependent mechanisms.

Increased production of ROS, which are primarily generated due to mitochondrial dysfunction during renal I/R injury, significantly contributes to tissue injury (Bhargava & Schnellmann, 2017; Ishimoto & Inagi, 2015). MDA, as an end product of lipid peroxidation induced by ROS, can directly cause cytotoxic damage (Buege & Aust, 1978; Gaweł et al., 2004). On the other hand, antioxidants such as SOD, CAT and GSH possess protective effects against oxidative stress (Blokhina et al., 2003). It is accepted that the imbalance between oxidants and anti‐oxidants can reflect the level of ROS metabolism and tissue damage (Singh et al., 1993). In the present study, we observed a significant increase in renal MDA and decrease in renal GSH level and SOD activity in I/R rats, while HNG administration downregulated MDA and upregulated SOD activity and GSH levels. Similarly to our findings, previous studies have suggested that HNG suppresses oxidative stress while enhancing the antioxidant defence system in I/R models involving various tissues including cardiac and cerebral tissues as well as in diverse cell types (Klein et al., 2013; Zhao et al., 2012). One of the proposed mechanisms underlying the anti‐oxidative effects of HNG is its ability to directly modulate mitochondrial function (Kumfu et al., 2016). It is proposed that HNG exerts this effect through mitochondrial colocalization and/or via activation of the STAT3 signalling pathway (Gao et al., 2017), which is consistent with our results (Figure 4b). Moreover, p‐STAT3 has also been reported to inhibit the formation of the mitochondrial permeability transition pore (mPTP), thereby preserving the mitochondrial membrane potential (ΔΨm), reducing the generation of ROS and suppressing apoptosis (Boengler et al., 2010).

In the present study HNG treatment did not improve CAT activity, which was reduced by I/R injury. During renal I/R, unlike other antioxidant enzymes, both CAT activity and CAT protein synthesis are known to decrease (Singh et al., 1993). The inability of HNG to enhance CAT activity observed in our study may be attributed to a reduction in CAT protein levels. Indeed, previous studies employing in experimental I/R models have demonstrated that HNG treatment preserves SOD and glutathione peroxidase activities in intestinal and brain tissues following I/R injury. However, CAT activity was not evaluated in those investigations (Abozaid et al., 2023; Zhao et al., 2012).

It has been verified that during renal I/R injury, inflammation is one of the most important mechanisms involved in tissue damage (Sharfuddin & Molitoris, 2011; Thurman, 2007). On the other hand, HNG has been characterized as an anti‐inflammatory agent due to its suppressive effects on pro‐inflammatory cytokines (Zhao et al., 2012). It has been shown that HNG reduces age‐related neuroinflammation and suppresses pro‐inflammatory cytokines in an intestinal I/R model, in diabetic nephropathy and traumatic brain injury (Abozaid et al., 2023; Moin et al., 2023; Thapak et al., 2024). In our study, to investigate the inflammatory response associated with renal I/R injury, we assessed MPO activity as a marker of neutrophil infiltration into kidney tissues. Consistent with previous findings, our results also demonstrated that HNG treatment significantly attenuated I/R‐induced inflammation in renal tissue.

In conclusion, to the best of our knowledge, this is the first study to demonstrate that HNG modulates oxidative stress, apoptosis, inflammation and mitochondrial function in a rat model of renal I/R‐induced AKI. The findings suggest that these beneficial cellular and molecular effects may be associated, at least in part, with the activation of the STAT3 and/or ERK1/2 signalling pathways (Figure 5). However, despite these improvements, HNG did not significantly ameliorate renal dysfunction or histopathological injury under the experimental conditions of the present study. These findings reveal a dissociation between cellular and molecular protection and short‐term functional and histopathological recovery. Therefore, while HNG shows promise as a modulator of key pathogenic pathways involved in renal I/R injury, further studies are needed to determine whether different treatment regimens or longer reperfusion periods can ultimately improve renal function and histopathological outcomes.

**FIGURE 5:**
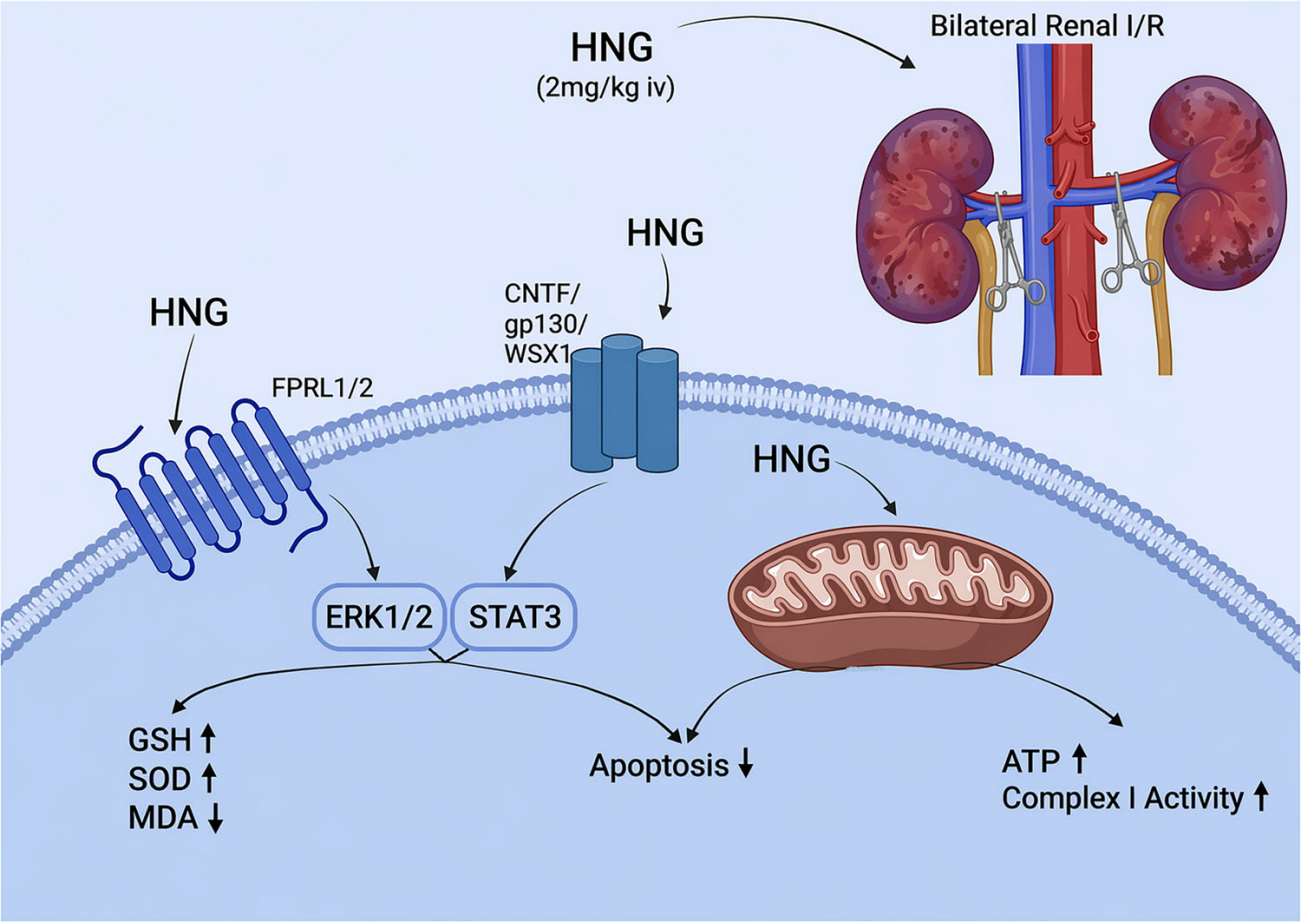
Schematic representation of the effects of HNG on mitochondrial function and oxidative stress following renal ischaemia–reperfusion injury. HNG treatment increased ATP levels and complex I activity, enhanced antioxidant defences by increasing GSH and SOD levels, and reduced MDA levels and apoptosis. These effects may be associated with activation of the ERK1/2 and STAT3 signalling pathways. CNTF, ciliary neurotrophic factor; ERK1/2, extracellular signal‐regulated kinases 1 and 2; FPRL1/2, formyl peptide receptor‐like 1/2; GSH, glutathione; HNG, humanin G; I/R, ischaemia–reperfusion; MDA, malondialdehyde; SOD, superoxide dismutase; STAT3, signal transducer and activator of transcription 3.

### 4.1 Limitations

The present study has several limitations that should be considered when interpreting the findings. The involvement of the STAT3 and ERK1/2 signalling pathways was inferred from changes in protein phosphorylation. Since pathway‐specific inhibition experiments were not performed, the present findings demonstrate an association rather than a causal mechanistic relationship. Western blot analyses were performed with a relatively small sample size, and these findings should therefore be interpreted with appropriate caution. In addition, the sample size differed among individual analyses because tissues obtained from the same animals were allocated to multiple experimental procedures, and the number of samples included in certain assays was limited by tissue availability and assay‐specific constraints. Furthermore, resource limitations precluded the inclusion of additional mechanistic experiments, such as pathway inhibition studies and more comprehensive mitochondrial analyses. Future studies using longer reperfusion periods and more extensive mechanistic approaches are warranted to further validate these findings.

## AUTHOR CONTRIBUTIONS

All authors have read and approved the final version of this manuscript and agree to be accountable for all aspects of the work in ensuring that questions related to the accuracy or integrity of any part of the work are appropriately investigated and resolved. Filiz Basrali and Leyla Abueid conceived and designed research. Leyla Abueid, Ahmet Faruk Torun and Ezgi Golal conducted the experiments. Filiz Basrali, Leyla Abueid and Nuray Acar analyzed the data. Filiz Basrali and Leyla Abueid wrote the manuscript. All persons designated as authors qualify for authorship, and all those who qualify for authorship are listed.

## CONFLICT OF INTEREST

None declared.

## GENERATIVE AI STATEMENT

During the preparation of this manuscript, the authors utilized OpenAI's ChatGPT and Google's Gemini models for language editing, grammar correction and assisting in the structuring of the manuscript text. The tools were used solely to refine prose, formatting and structural clarity. All primary data collection, statistical analyses, interpretation of results and final scientific content were conducted, reviewed and verified exclusively by the authors. The final version was fully approved by all authors.

## Data Availability

The corresponding author is willing to provide the data and datasets used and/or analysed during the current study upon reasonable request.
